# APOL1 is a novel prognostic biomarker in thyroid cancer and correlates with immune infiltration

**DOI:** 10.3389/fonc.2025.1707078

**Published:** 2025-11-25

**Authors:** Jianting Man, Xingru Tao, Zongyuan Zhang, Tiefeng Shi

**Affiliations:** 1 The Fourth Department of General Surgery, The Second Affiliated Hospital of Harbin Medical University, Harbin, Heilongjiang, China; 2 School of Stomatology, Southwest Medical University, Luzhou, Sichuan, China

**Keywords:** apolipoprotein 1, thyroid cancer, immune infiltration, prognostic marker, bioinformatics

## Abstract

**Background:**

Although apolipoprotein 1 (APOL1) expression is upregulated in several types of cancers, including thyroid cancer (THCA), its specific role in THCA remains unclear. The aim of the present study was to analyze whether APOL1 could serve as a prognostic marker and predictor of immune cell infiltration (ICI) in THCA.

**Methods:**

APOL1 expression was assessed using TCGA and GTEx data, based on data from The Cancer Genome Atlas (TCGA). The diagnostic and prognostic value of APOL1 was assessed using Kaplan-Meier survival curves, receiver operating characteristic curves, Cox regression analysis and enrichment analysis in R. Kyoto Encyclopedia of Genes and Genomes and Gene Ontology were used to determine APOL1-related pathways. Furthermore, the correlation between APOL1 expression and ICI, as well as immune checkpoint genes, was investigated. Western blotting, cell counting Kit-8, wound healing assays, Transwell assays and 5-EdU were used to examine APOL1 expression and function in THCA.

**Results:**

Abnormally elevated expression of APOL1 was observed in THCA tissues based on the data from TCGA and GTEx. Functional biological analysis indicated a strong connection between APOL1 and immune signatures. Additionally, APOL1 expression showed a correlation with infiltration of various immune cell types, including macrophages, neutrophils, dendritic cells, T and B cells, Th1/2 cells, CD8 T cells and Tregs. The results of western blotting confirmed upregulated APOL1 expression in THCA tissues. *In vitro* experiments showed that knockdown of APOL1 promoted THCA proliferation, invasion and migration. APOL1 serves as a valuable prognostic biomarker and is related to disease progression and ICI in THCA.

**Conclusions:**

In this study, we innovatively explored the potential association between APOL1 and the immune microenvironment, and demonstrated that APOL1 expression is upregulated in THCA tissues. Knockdown of APOL1 in THCA cells results in promoted proliferation and migration. Additionally, APOL1 may regulate ICI in the THCA microenvironment.

## Introduction

Thyroid cancer (THCA) represents the most widespread endocrine cancer globally, and its incidence has increased in recent decades, Global Cancer Statistics 2022 reported that THCA ranks 7^th^ in global cancer incidence (1). Papillary thyroid carcinoma (PTC) represents the predominant pathological subtype among THCA, accounting for approximately 85%–90% of all THCA cases (2, 3). While treatment modalities like surgery, radiation therapy, levothyroxine administration and targeted therapy typically yield favorable outcomes, ~10% of patients with aggressive PTC develop local recurrence or a distant metastasis within 10 years, highlighting challenges in diagnosing and managing metastatic recurrence in advanced cases (4–7). The establishment of the tumor microenvironment (TME) fosters interactions between immune cells and the tumor, thwarting immune system destruction (8). Moreover, tumor progression and invasion can be facilitated by inflammatory factors secreted by immune cells and/or tumor cells (9). Additionally, numerous immune cells exhibit high expression levels in THCA and are distributed in the TME. Therefore, identifying potential tumor markers may enhance our comprehension of the molecular underpinnings of THCA, aiding in its diagnosis and the development of targeted therapies.

Apolipoprotein L1 (APOL1), located on chromosome 22q12.3 and encoded by the APOL1 gene, exists as two variants: APOL1G1/2 (10, 11). APOL1 plays a crucial role in protecting humans against trypanosome infections (12) and is associated with genetic variations linked to non-diabetic kidney disease (10). APOL1, a secreted high-density lipoprotein (HDL), binds to apolipoprotein A1 (APOA1), which is a key player in lipid transport and metabolism (11). Increasing evidence indicates that apolipoproteins are implicated in various biological processes, encompassing inflammation, immune response and tumor progression (13–15). The direct involvement of apolipoproteins has been documented in cell proliferation, apoptosis, migration and invasion across various types of cancer (16, 17). APOL1 exhibits overexpression in a range of tumors, including head-and-neck squamous cell carcinoma (HNSC) and hepatocellular carcinoma (18, 19). Xiao et al (20) reported that elevated APOL1 expression was correlated with a worse clinical prognosis, promoting clear-cell renal cell carcinoma proliferation, metastasis and xenograft tumor formation. Lin et al (21) investigated APOL1 overexpression promotes proliferation and suppresses apoptosis in pancreatic cancer. Chidiac et al (22) demonstrated APOL1 overexpression in PTC; however, the underlying biological function remains elusive.

To date, only a limited number of biomarkers have been identified as useful for the diagnosis and prognosis of THCA. However, many of these biomarkers are challenging to detect initially. Shukha et al (23) confirmed that the liver is the primary source of circulating APOL1, offering a more accessible method for detecting APOL1 as a prognostic biomarker. This study elucidates the prognostic roles of APOL1 expression in THCA. Subsequently, functional enrichment analysis of APOL1 was performed using Gene Ontology (GO), Kyoto Encyclopedia of Genes and Genomes (KEGG) and immune cell infiltration (ICI) analysis. Furthermore, knockdown of APOL1 promoted PTC cell proliferation and invasion *in vitro*. Collectively, it was shown that APOL1 was associated with ICI and was pivotal in THCA development, potentially serving as a prognostic biomarker for patients with THCA.

## Materials and methods

### APOL1A differential expression in THCA

RNA-seq data from cancer types were acquired from TCGA (https://portal.gdc.cancer.gov/) and GTEx (https://gtexportal.org/). HTSeq-FPKM and HTSeq-Count data for THCA samples were also obtained from TCGA (https://portal.gdc.cancer.gov/repository) for subsequent analysis. The present study fully adhered to TCGA and GTEx guidelines. APOL1 expression across various pathological stages of THCA was determined using the GEPIA database (http://gepia.cancer-pku.cn/index.html). At the same time, the clinical data of 512 cases of thyroid cancer sequenced from the TCGA database were used for subsequent analysis.

### Expression and enrichment analysis of APOL1 correlated genes, analysis of differentially expressed genes and protein-protein interaction network generation

LinkedOmics (http://www.linkedomics.org/) (24) was used to assess the top 50 genes positively or negatively correlated with APOL1 and to produce a heatmap, selecting genes with an adjusted P-value (adj P) <0.05 for functional enrichment analysis. Metascape (https://metascape.org) (25) was used to visualize the enriched biological processes (BP), cellular components (CC), molecular functions (MF) and KEGG pathway terms associated with APOL1 and its co-expressed genes.

The DESeq2 R package was used to compare APOL1 expression data between low and high expression groups (using a cut-off value of 50%) in THCA samples, to identify DEGs (26). Employing the R package ggplot2 (version 3.3.6), volcano plots were plotted. The prediction of the PPI network of DEGs was performed using the Search Tool for the Retrieval of Interacting Genes (STRING) database (27), setting an interaction score threshold of 0.4 as the cut-off criterion. Meanwhile, PPI network visualization was performed using Cytoscape (version 3.7.1), identifying the most significant modules within the network with MCODE (version 1.6.1) (28). DEGs with a threshold of |log fold change (logFC)|>2 and an adj P <0.05 were used for functional enrichment analysis employing the Cluster Profiler package in R (29).

### Correlation analysis of APOL1 expression and clinicopathological features of patients with THCA

Receiver operating characteristic (ROC) curves were used to compare APOL1 expression levels between tumor and normal tissues, evaluating its predictive value for THCA diagnosis. A Wilcoxon rank-sum test (for continuous variables) or Spearman χ^2^ test (for ranked variables) was used to compare clinicopathological characteristics between groups with high and low APOL1 expression. Logistic regression analysis was used for correlation assessment between APOL1 expression and clinicopathological features. Prognostic analysis was performed using Kaplan-Meier (K-M) analysis, along with univariate and multivariate Cox regression analyses.

### Generation and prediction of prognostic models

The patients were categorized into high and low groups based on the median APOL1 expression levels. Univariate Cox regression analysis was performed and K-M survival curves were plotted using survminer (version 0.4.9) and survival packages (version 3.2-10) to uncover genes that are correlated with prognosis in the two groups. Subsequently, the glmNet (version 4.1-2) and survival packages were used for analysis of the association of APOL1 expression with the top 100 positively and negatively correlated genes by seven-fold cross-validation for prognostic Least Absolute Shrinkage and Selection Operator (LASSO) coefficient screening. The final signature of T-cell receptors was determined through further screening of risk characteristics, calculating the risk score as follows: Risk score = Σ(expression level of each gene x correlation coefficient). To personalize the overall survival (OS) prediction in patients with THCA, a nomogram was generated using the R package, RMS (version 5.1-3). The nomogram was comprised of genes screened through LASSO coefficient filtering, significant clinical features and calibration plots. The Concordance index (C-index) was used to assess the discriminative ability of the nomogram.

### Analysis of ICI and expression of immune checkpoints

The immune infiltration correlation analysis of APOL1 was conducted using ssGSEA and ESTIMATE packages within the GSVA package (version 1.34.0). The correlation between APOL1 expression and immune checkpoint expression in THCA was evaluated using a Wilcoxon rank-sum test. The association between expression of APOL1 and the immune checkpoint was assessed using a Spearman χ^2^ test.

### Cell culture, RNA interference transfection and tissue samples

KTC-1 and TPC-1 Human THCA cells were provided by Suzhou Haixing Biosciences Co., Ltd., and were cultured in 1640 (cat. no. MA0212, MeilunBio) supplemented with 10% FBS (cat. no. 164210, Pricella) and 1% penicillin-streptomycin at 37˚C with 5% CO_2_. The APOL1-siRNA (si-APOL1: Sense: 5’-GCAGUACAGAAACUGGUUUTT-3’; Anti-sense e: 5’-AAACCAGUUUCUGUACUGCTT-3’) and negative control siRNA (si-NC Sense:5’-UUCUCCGAACGUGUCACGUTT-3’; Anti-sense:5’-ACGUGACACGUUCGGAGAATT-3’) were purchased from GenePharma (Shanghai,China). Transfection with si-RNAs was performed using Lipofectamine 8000 (Beyotime Institute of Biotechnology) according to the manufacturer’s protocol. The PTC and adjacent non-cancerous thyroid tissue samples were gathered from the Thyroid Surgery Department of the Second Affiliated Hospital of Harbin Medical University. The selection criteria included patients diagnosed with thyroid cancer before surgery and those who have never received any treatment from February 2024 to May 2025. The study was conducted in strict accordance with the Declaration of Helsinki and was approved by the Ethics Committee of the Second Affiliated Hospital of Harbin Medical University (No. KY2024-037).

### RT-qPCR

Total RNA was extracted using TRIzol reagent (Invitrogen, USA) according to the manufacturer’s instructions. Complementary DNA was synthesized at 42 °C using a reverse transcription kit (Takara Biotechnology Co., Ltd., Dalian, China). Quantitative real-time PCR was performed with SYBR Green Supermix (Bio-Rad Laboratories, USA) following a standard two-step amplification protocol. GAPDH served as the internal reference gene for normalization of mRNA expression levels. Relative gene expression was calculated using the 2^−ΔΔCt method. All primers were synthesized by Sangon Biotech Co., Ltd. (Shanghai, China), with the following sequences: APOL1 F: 5′- TCAACCCCTCTTTTCCTGCTC -3′, APOL1 R: 5′-CGAGGGGCTTACTTTGAGGA-3′; GAPDH F: 5′-CTGGGCTACACTGAGCACC-3′, GAPDH R: 5′-AAGTGGTCGTTGAGGGCAATG-3′.

### Western blot

Total protein was extracted using RIPA lysis buffer containing protease inhibitors. Protein concentration was determined using a bicinchoninic acid assay (Beyotime Institute of Biotechnology). Next, proteins were resolved by SDS-PAGE on 10% SDS gels and subsequently transferred to PVDF membranes (MilliporeSigma). The membrane was blocked with 5% milk for 2 hours at room temperature, and then subsequently incubated with specific primary antibodies targeting APOL1 (Abcam, cat. no. ab108315) at 4 °C overnight. The secondary antibody was then incubated for 1 hour at room temperature (Anti-rabbit IgG, HRP-linked Antibody #7074). The signal intensity was analyzed using a LI-COR Odyssey Imaging System (LI-COR Biosciences) according to the manufacturer’s protocol.

### Wound healing, Transwell migration and invasion, cell viability and cell proliferation assays

Cells (5x10^5^ cells per well) were seeded into a 6-well plate and incubated at 37˚C overnight. Subsequently, a 200 μl pipette tip was used to create a scratch in the monolayer of cells and the cells were gently washed with PBS. The scratch area was immediately imaged and then incubated for 24 h, after which, the same area was imaged using a bright field microscope.

For the Transwell assay, 1*10^4^ cells were seeded in 24-well Transwell insert chambers (Corning, Inc.), pre-coated with 2% Matrigel for the invasion assay or without Matrigel for the migration assays, in serum-free medium. In the lower chamber, media supplemented with 15% FBS was added to the lower. After 2 days, the cells that had not migrated were removed, and those that had successfully traversed were fixed and stained using crystal violet (Kaigen, China) for 15 min. T. Cells were counted using a microscope.

Cell viability was assessed using CCK-8 and EdU assays (CCK-8, cat. no. MA0218, MeilunBio). Cells were seeded in 96-well plates (3x10^3^ cells per well) and cultured in a humidified incubator, with five wells per condition. After 0, 1, 2, 3 and 4 days of culture, 10 μl CCK-8 reagent was added to each well and cells incubated for 3 h, after which, the absorbance was measured at 450 nm. Each assay was independently conducted in triplicate. To assess cell proliferation, a BeyoClick™ EdU-488 cell proliferation kit (Beyotime Institute of Biotechnology) was used according to the manufacturer’s protocol. The treated cells were observed using a fluorescence photomicroscope. The number of fluorescent cells was counted in at least three random fields of view.

### Statistical analysis

In this study, the APOL1 cut-off value was determined by ranking all samples according to APOL-1 expression levels, with the top 50% representing high expression and the bottom 50% representing low expression. Data are presented as the mean ± standard deviation (SD) of three repeats, and were analyzed using GraphPad Prism 9 (Dotmatics) or R version 4.1.2. P<0.05 was considered to indicate a statistically significant difference.

## Results

### Differential expression of APOL1 in THCA

**Figure 1:**
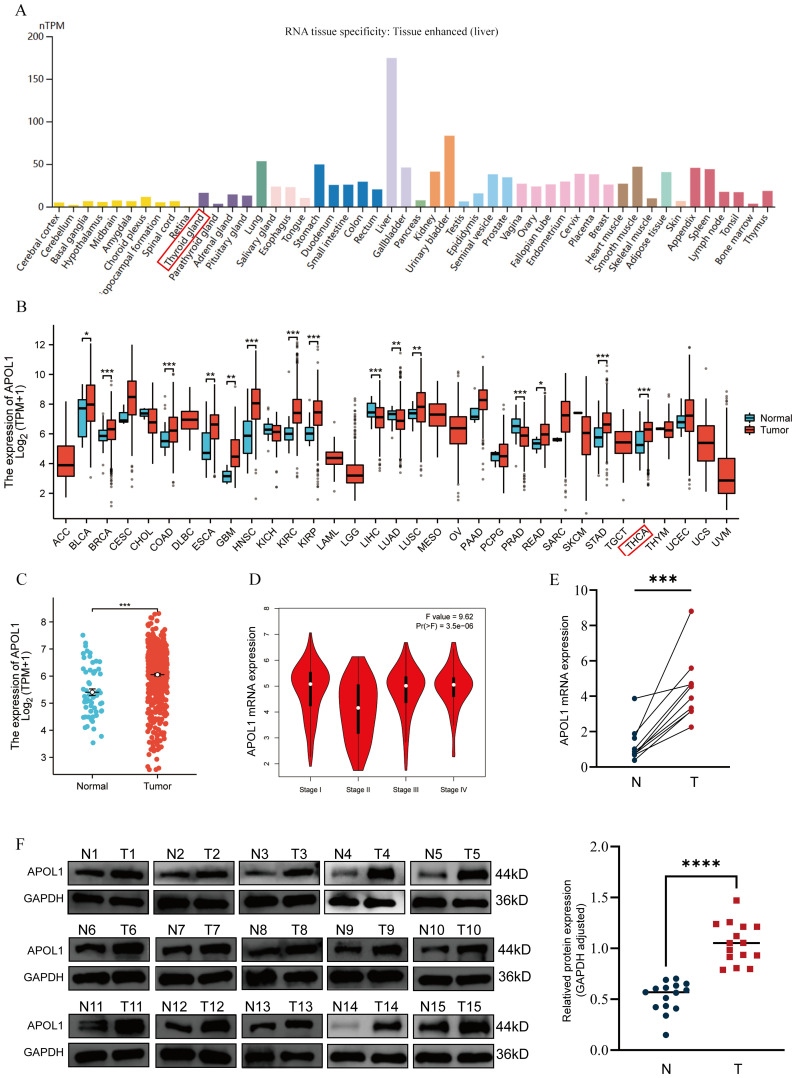
APOL1 expression in tissues and THCA. **(A)** APOL1 mRNA levels across various cancer types and corresponding normal tissues. **(B)** Pan-cancer analysis of APOL1 expression. **(C)** APOL1 expression in THCA. **(D)** APOL1 levels across different pathological stages of THCA. **(E)** APOL1 mRNA expression levels in tumors and thyroid tissues. **(F)** Western blot analysis showed upregulated APOL1 expression in tumor tissues compared with the normal thyroid tissues. *P < 0.05, **P < 0.01, ***P < 0.001, ****P < 0.0001.

Analysis of the HPA database revealed no notable difference in APOL1 expression across tissues, with the exception of the liver (**Figure 1A**). Using RNA-seq data from TCGA and GTEX, APOL1 expression was analyzed in various tumor tissues compared to the respective normal tissues. The results indicated significantly higher APOL1 levels in breast invasive carcinoma, colon adenocarcinoma, HNSC, kidney renal clear cell carcinoma, kidney renal papillary cell carcinoma, stomach adenocarcinoma and THCA (**Figures 1B, C**). Analysis using the GEPIA database showed a significant downregulation of APOL1 in stage II THCA, prompting further investigation of the role of APOL1 in THCA (**Figure 1D**). Next, APOL1 expression was assessed in 25 THCA tissues and their corresponding non-cancerous tissues, 15 of which were used for western blotting experiments and 10 for PCR analysis. The results showed that APOL1 was upregulated in cancer tissues compared with adjacent normal tissues (**Figures 1E, F**). Clinical baseline data are shown in **Supplementary Table S1**.

### Enrichment analysis of APOL1 and co-expressed genes in THCA

**Figure 2:**
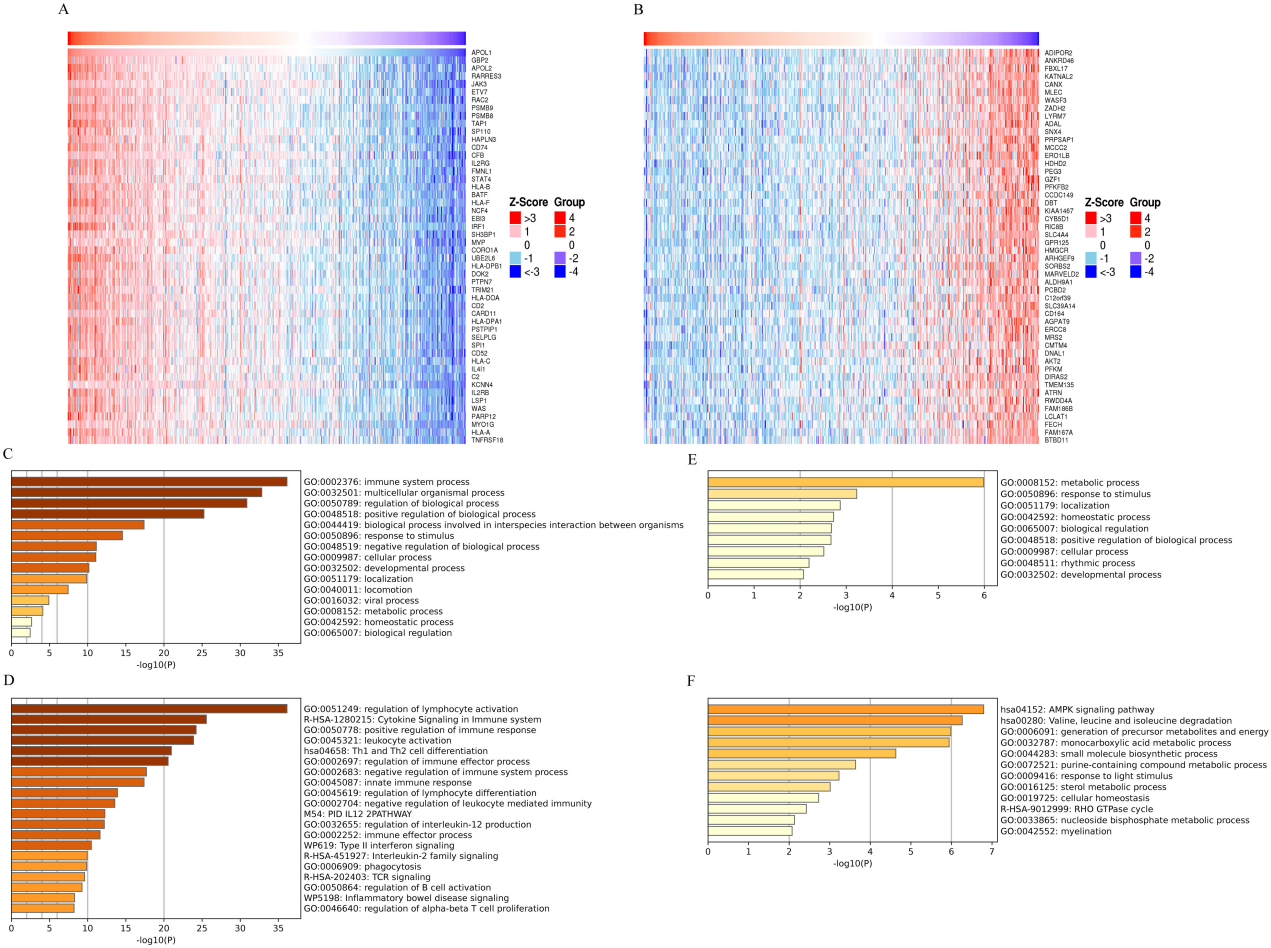
GO and KEGG analysis of APOL1 and co-expressed genes in THCA. **(A, B)** Heatmaps display the top 50 genes **(A)** positively and **(B)** negatively correlated with APOL1 in THCA. **(C, D)** GO and KEGG pathway terms for genes **(E)** positively and **(F)** negatively correlated with APOL1.

To investigate the APOL1-affected functions and pathways, TCGA data were used to identify genes positively or negatively co-expressed with APOL1 in THCA. A heatmap (**Figures 2A, B**) was used to display the top 50 genes positively and negatively correlated with APOL1. Potential functional pathways of these genes were then analyzed using the Metascape database. Notably, GO enrichment analysis of BP revealed that genes positively associated with APOL1 were predominantly related to the “Immune system process” (**Figure 2C**). Additionally, the KEGG pathways involving APOL1 and its correlated genes are shown in **Figure 2D**. Pathways enriched included “Cytokine Signaling in the Immune system” and “Th1 and Th2 cell differentiation”. The GO and KEGG pathway results for genes negatively related to APOL1 are shown in **Figures 2E, F**. These findings suggest a strong association between high APOL1 expression and immune processes in THCA. Gene expression profiles for groups with high and low APOL1 expression were analyzed, aiming at identifying differences in median mRNA levels. A total of 206 statistically significant DEGs were analyzed between the high and low APOL1 expression groups (|logFC| >2, adj P<0.05), including 116 up-regulated and 90 down-regulated genes. **Supplementary Figure S1** shows a heatmap with the top 10 positively and negatively correlated genes with APOL1. Moreover, both up-regulated and down-regulated genes were significantly enriched. A PPI network for common DEGs was constructed and hub genes were screened.

### Association of APOL1 expression with clinical features in THCA

**Figure 3:**
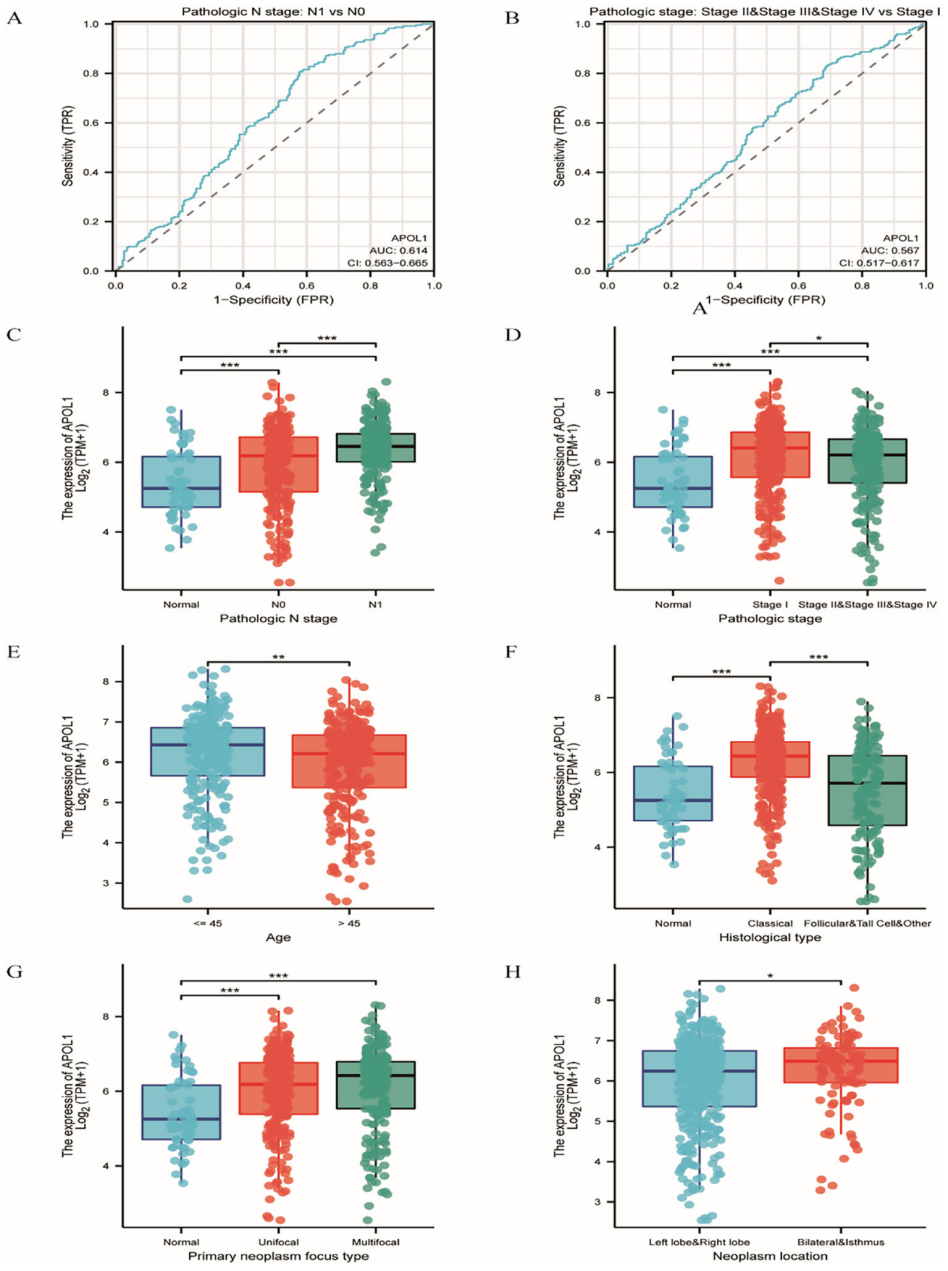
Association of APOL1 expression with clinical features in THCA. **(A, B)** ROC curves for APOL1 in evaluating diagnostic efficacy of pathologic stage and extrathyroidal extension in THCA. **(C–H)** Associations of APOL1 expression with pathologic N Stage, overall pathologic stage, age, histological type, primary neoplasm focus type and neoplasm location in THCA. *P < 0.05, ***P < 0.001.

To investigate the clinical characteristics associated with APOL1 in THCA, patients were categorized into low and high APOL1 expression groups (**Supplementary Table S1**). The median APOL1 expression, calculated as log_2_(TPM + 1), served as the cutoff value. Correlation analysis indicated significant associations between APOL1 expression and variables, including pathologic N stage, overall pathologic stage, age, histological type, primary neoplasm focuses type and neoplasm location (P<0.05; **Supplementary Table S2**). APOL1 upregulation was positively correlated with several clinicopathological features including pathologic N stage (OR 1.874; P<0.001), overall pathologic stage (odds ratio (OR) 0.638; P = 0.013), age (OR 0.614; P = 0.006), histological type (OR 0.388; P<0.001), primary neoplasm focus type (OR 0.658; P = 0.02) and neoplasm location (OR 0.590; P = 0.016). Logistic regression was used to further validate the link between THCA clinicopathological factors and APOL1 expression levels. ROC curve analysis was performed to evaluate APOL1 as a potential biomarker in patients with THCA at different N and pathologic stages, resulting in area under the curve (AUC) values of 0.614 and 0.567, respectively (**Figures 3A, B**). Additionally, the Wilcoxon Rank Sum was used to compare APOL1 expression across the various clinicopathological features. The results indicated significantly higher APOL1 expression in patients with Pathologic N stage N1, stages II-IV, age >45, classical type, unifocal or multifocal, and bilateral or isthmus tumors (**Figures 3C–H**; P<0.05).

### APOL1 as a prognostic biomarker for the prediction of survival in patients with THCA

**Figure 4:**
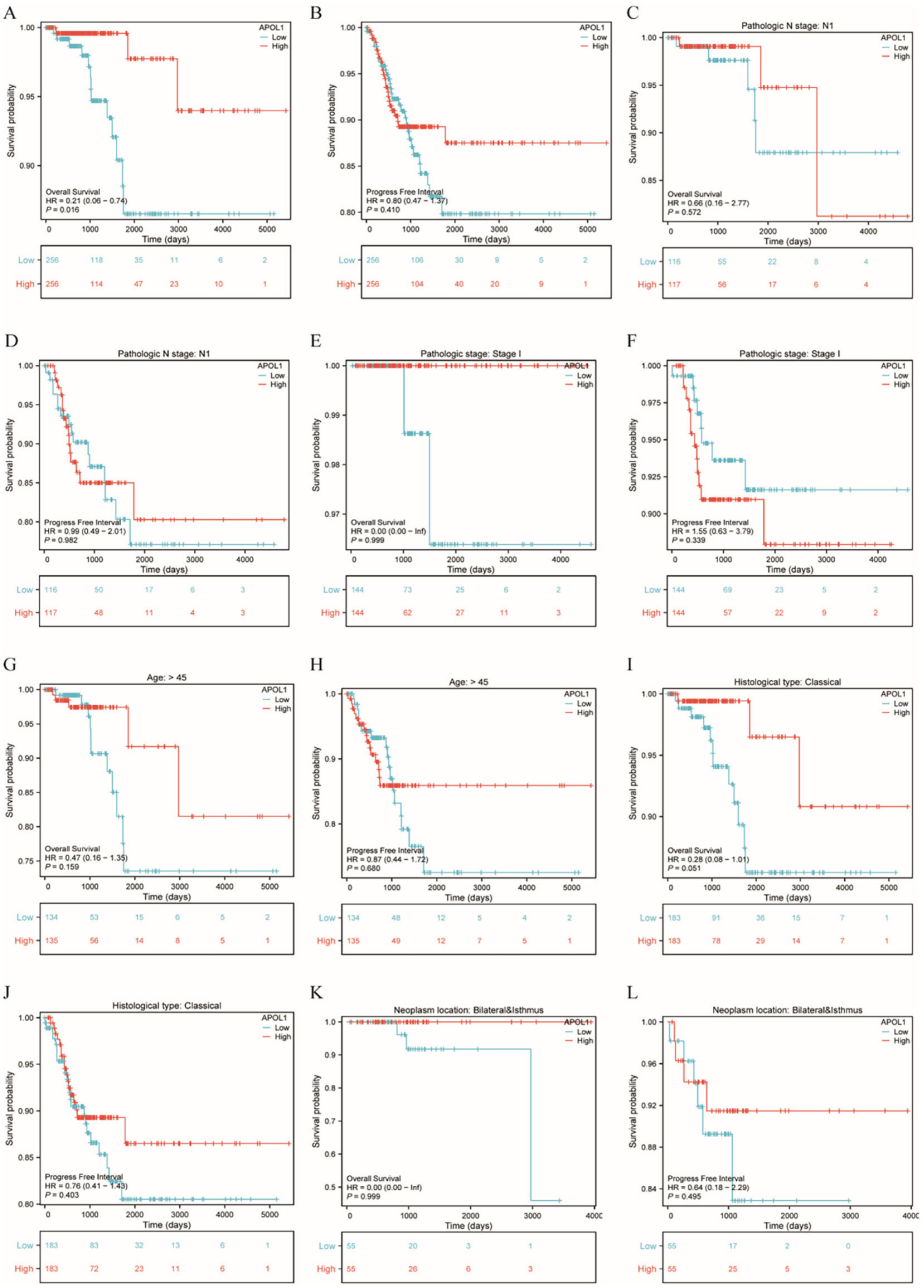
Prognostic analysis of APOL1 in patients with THCA. **(A, B)**: Kaplan-Meier (K-M) Curves: Overall Survival (OS) and PFI in THCA. **(C, D)** K-M curves showing the OS and PFI in patients with N1 pathological stage. **(E, F)** K-M curves showing the OS and PFI in patients with THCA with T1 pathological stage. **(G, H)** K-M curves showing the OS and PFI of patients aged over 45. **(I, J)** K-M curves showing the OS and PFI of patients with classical histological type. **(K, L)** K-M curves showing the OS and PFI of patients with THCA with neoplasms located bilaterally and in the isthmus.

K-M analysis was used to determine the correlation between APOL1 expression and prognosis in patients with THCA. Patients with high APOL1 expression exhibited a longer OS (hazard ratio (HR)=0.21 (0.06-0.74), P = 0.016) and a similar progression-free interval (PFI, HR = 0.80 (0.47-1.37), P = 0.41) than those with low expression (**Figures 4A, B**). K-M analysis showed that APOL1 upregulation was associated with a favorable prognosis in patients with Pathologic N stage N1 (OS, HR = 0.66 (0.16-2.77), P = 0.572; PFI, HR = 0.99 (0.49-2.01), P = 0.982), age >45 (OS, HR = 0.47 (0.16-1.35), P = 0.159; PFI, HR = 0.87 (0.44-1.72), P = 0.680) and classical histological type (OS, HR = 0.28 (0.08-1.01), P = 0.051; PFI, HR = 0.76 (0.41-1.43), P = 0.403) (**Figures 4C–L**).

### Development of a prognostic model for APOL1 in THCA

**Figure 5:**
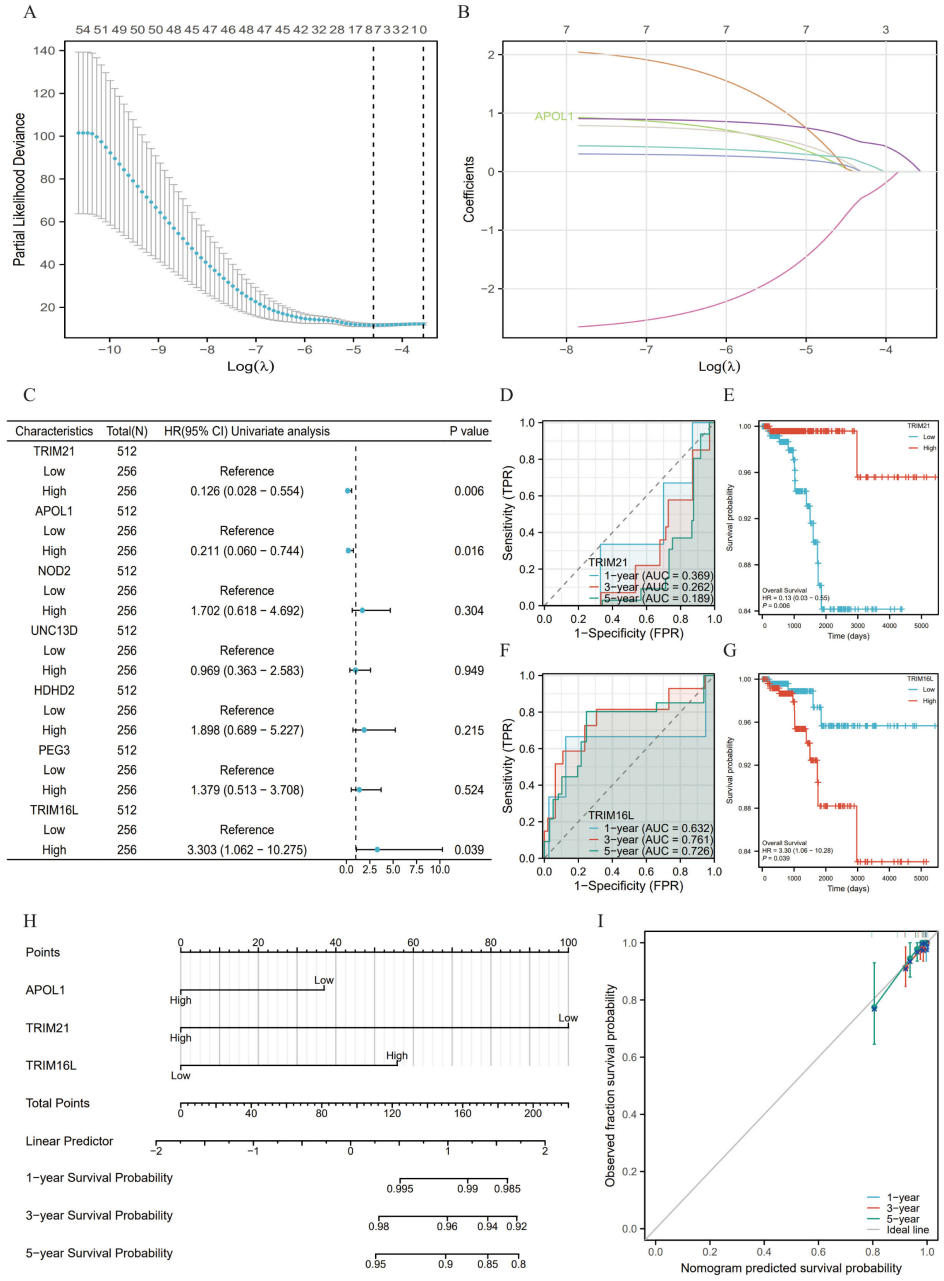
Prognostic model of APOL1 in THCA. **(A)** A total of seven genes were identified using LASSO regression coefficients. **(B)** Trajectories of variables selected by LASSO regression. **(C)** Forest plot of the Cox regression results for seven genes. **(D, F)** Time-dependent ROC curves for TRIM21 and TRIM16L expression. **(E, G)** Kaplan-Meier survival curves for all the patients with THCA categorized based on TRIM21 and TRIM16L expression levels. **(H)** A nomogram was used to predict the 1-, 3- and 5-year OS probabilities in THCA. **(I)** Calibration plot of the nomogram for the 1-, 3- and 5-year OS probabilities.

A total of 7 genes related to APOL1 were screened through LASSO regression to identify non-zero variables satisfying the lamb amin criterion. Risk score calculation was performed as follows: Risk score = 0.118*APOL1-0.892*TRIM21 + 0.231*NOD2 + 0.130*UNC13D+0.2*HDHD2 + 0.251*PEG3 + 0.635*TRIM16L (**Figures 5A, B**). The genes underwent multifactorial Cox regression analysis utilizing the survival package (**Supplementary Table S3**; **Figure 5C**). The predictive capability of the OS risk score was assessed over time using ROC curves. For high TRIM21 expression, the AUC values were 0.369, 0.262 and 0.189 (**Figure 5D**), and 0.632, 0.761 and 0.726 for high TRIM16L expression (**Figure 5F**) at 1, 3 and 5 years, respectively. K-M curves showed that high TRIM21 expression was a favorable prognostic factor (**Figure 5E**), whereas high TRIM16L expression was associated with a poor prognosis (**Figure 5G**). Additionally, multifactorial Cox regression analysis of THCA clinical characteristics was performed using the survival package (**Supplementary Table S4**). A nomogram was constructed based on the results of the Cox regression analysis using the RMS package, incorporating APOL1, TRIM21 and TRIM16L expression (**Figure 5H**), calculating the >50% survival probability for 1, 3 and 5 years. The nomogram calibration curve for OS showed predictive results aligning with observational data across all patients (**Figure 5I**).

### Association between APOL1 expression and ICI in THCA

**Figure 6:**
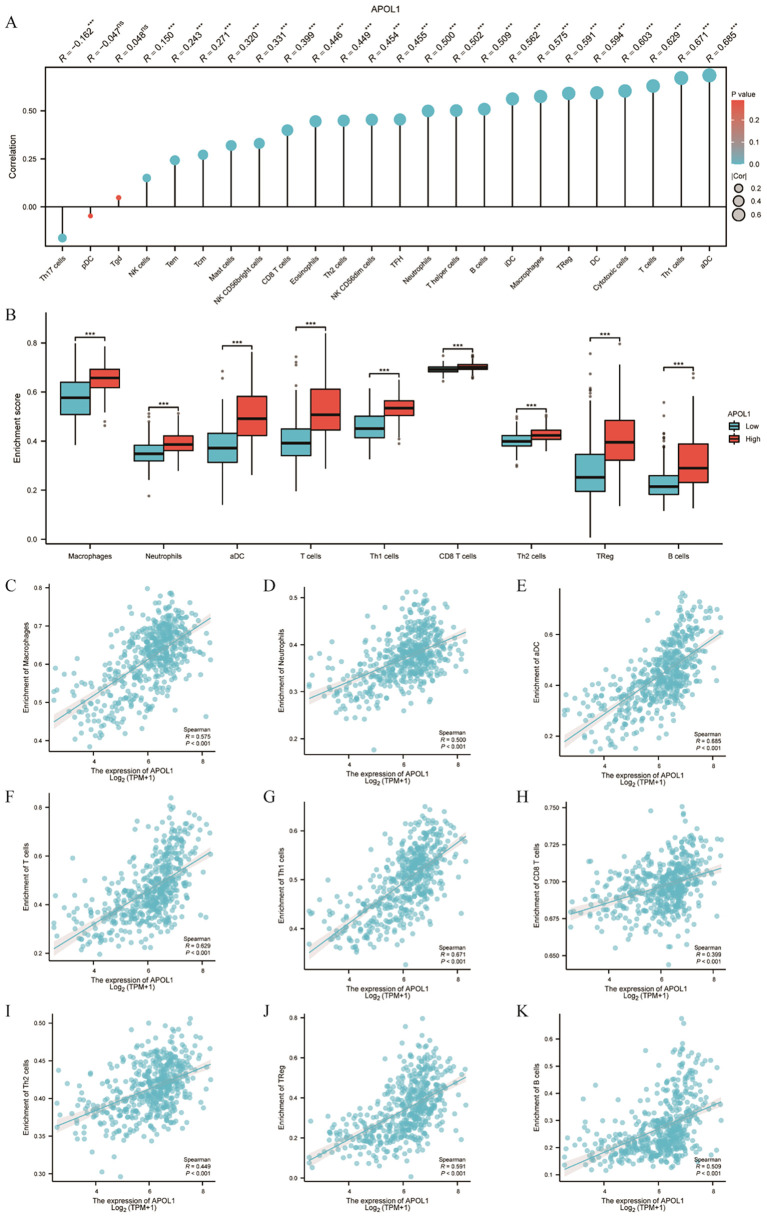
Association of APOL1 with immune infiltration in THCA. **(A)** Lollipop chart showing the positive correlation between APOL1 expression and various immune cells. **(B)** Correlation between APOL1 expression levels and the relative enrichment scores of macrophages, neutrophils, DCs, T and B cells, Th1/2, CD8+ T cells and Tregs. **(C–K)** Comparison of ICI of macrophages, neutrophils, DCs, T cells, Th1/2, CD8+ T cells, Treg and B cells between the high and low APOL1 expression groups. ***P < 0.001.

Spearman correlation analysis demonstrated that APOL1 expression in the THCA microenvironment was associated with ICI levels, as determined by SSGSEA. Specifically, APOL1 was positively correlated with various immune cells, including macrophages, neutrophils, DCs, T and B cells, Th1/2, CD8+ T cells and Tregs (**Figures 6A–K**). The findings indicated that APOL1 potentially promoted ICI and exerted immunomodulatory effects in THCA.

### Association between APOL1 expression and immune checkpoints in THCA

**Figure 7:**
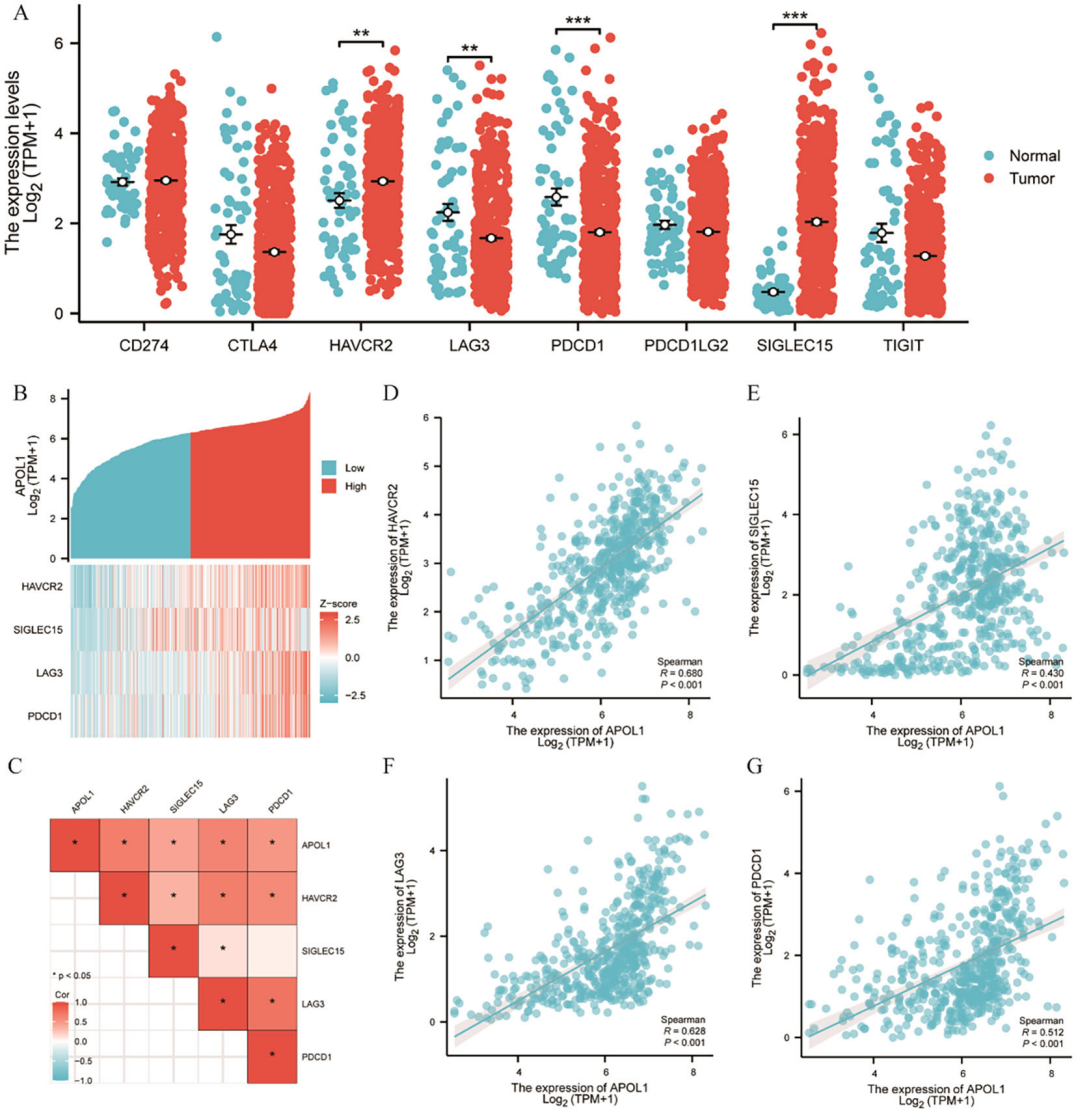
Correlation of APOL1 expression with immune checkpoints in THCA. **(A)** Spearman correlation analysis of APOL1 and immune checkpoint expression in THCA. **(B)** Heat map of APOL1 and immune checkpoint co-expression in THCA. **(C)** Heat map of the correlation between APOL1 expression and immune checkpoints. **(D, G)** Correlations of expression between APOL1 and the immune checkpoint genes HAVCR2, LAG3, PDCD1 and SIGLEC15. **P < 0.01, ***P < 0.001.

CTLA-4 and PD1/PD-L1 are crucial immune checkpoints facilitating tumor immune escape. The bioinformatics analysis demonstrated a correlation between APOL1 expression and ICI in THCA. Given its potential role in THCA, the association between APOL1 and immune checkpoints such as CTLA-4 and PDCD1 was investigated. The results identified HAVCR2, LAG3, PDCD1 and SIGLEC15 as significant immune checkpoints (**Figure 7A**). Co-expression with APOL1 was also analyzed (**Figure 7B**). Heat map analysis was used to explore the correlation between APOL1 expression and immune checkpoints, revealing a positive correlation with all four significant checkpoints (**Figure 7C**). Further Spearman analysis confirmed these findings, as shown in **Figures 7D–G**, aligning with the results in **Figure 7C**. Therefore, APOL1 may exert a regulatory or protective role in THCA by promoting the overexpression of certain immune checkpoints.

### Knockdown of APOL1 promotes migration and proliferation of PTC cells *in vitro*

**Figure 8:**
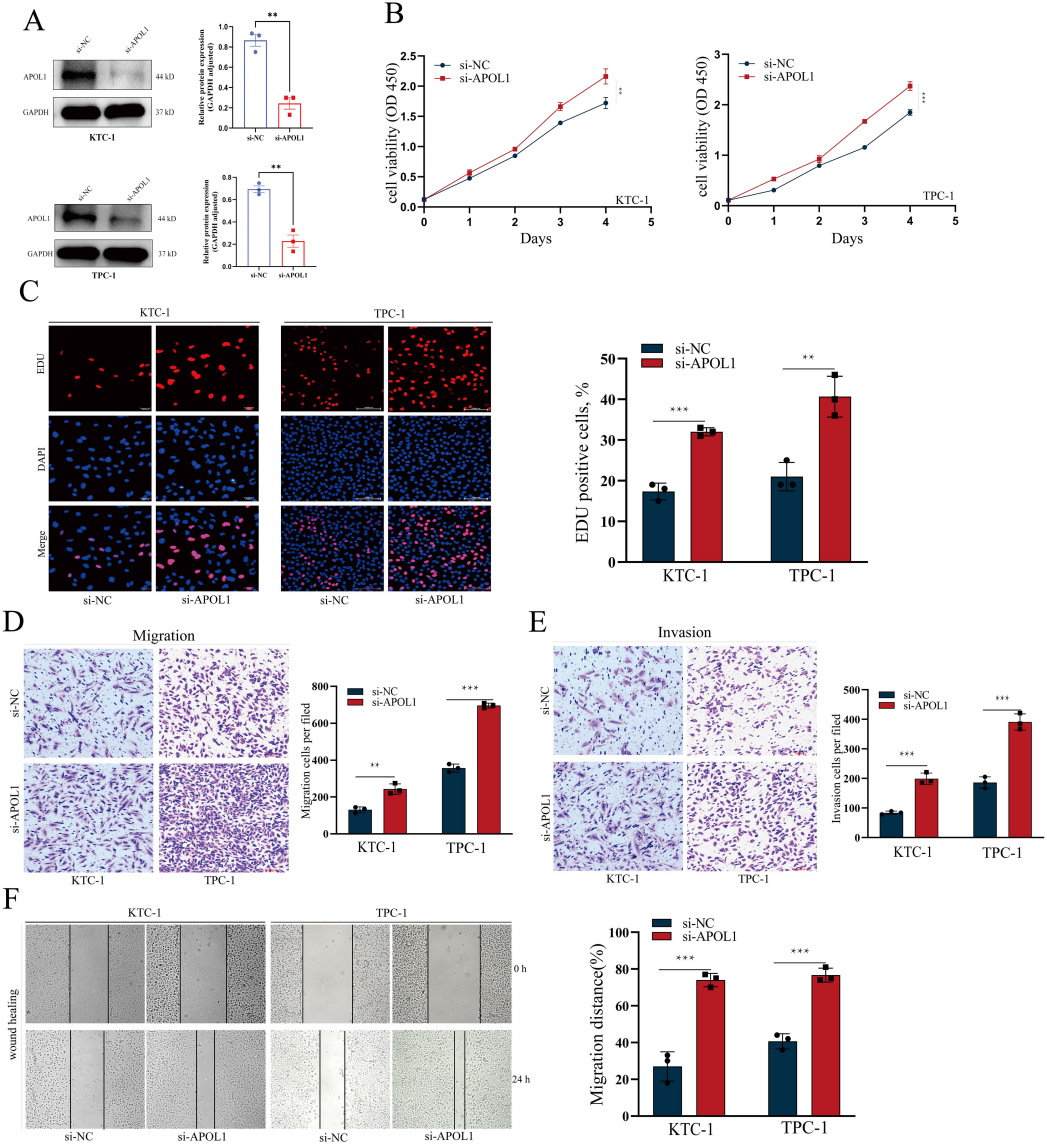
APOL1 knockdown enhances the proliferation and migration in PTC Cells. **(A, B)** Western blot analysis showing APOL1 knockdown efficiency in KTC-1 and TPC-1 cells. **(C–F)** CCK-8 and EdU assays assessing the proliferation of KTC-1 and TPC-1 cells following APOL1 knockdown. **(G–J)** Wound healing, Transwell migration and Transwell invasion assays were used to migratory ability of KTC-1 and TPC-1 cells following silencing APOL1. **P < 0.01, ***P < 0.001.

TPC-1 and KTC-1 cells were chosen for *in vitro* loss-of-function studies. Knockdown efficiency was verified by western blot analysis (**Figure 8A**). Following APOL1 knockdown, cell proliferation was assessed using CCK-8 and EdU incorporation assays. Importantly, APOL1 knockdown significantly promotes the proliferative capacity of PTC cells (**Figures 8B, C**). Moreover, Transwell and wound healing assays were employed to evaluate the role of APOL1 in PTC cell migration and invasion. The results indicated that APOL1 knockdown markedly increases both the speed of cell migration and the invasive capacity (**Figures 8D–F**). Together, APOL1 knockdown promoted PTC cell proliferation and migration.

## Discussion

THCA, the most prevalent malignant endocrine neoplasm, is gaining attention due to its rapidly increasing incidence worldwide (30). Currently, the standard treatment for THCA typically involves surgery, TSH suppression therapy and radioiodine therapy. However, radioiodine therapy may be ineffective against local recurrences and/or distant metastases (31). Accordingly, revealing promising diagnostic and prognostic biomarkers and demonstrating the mechanisms behind THCA progression is crucial.

In the present study, analysis of TCGA data revealed high APOL1 expression in 14 cancer types, notably in THCA, where it was significantly upregulated compared to normal tissue. Analysis of TCGA datasets demonstrated a close correlation between APOL1 expression and clinical as well as prognostic outcomes in THCA, including variables such as pathologic N stage, overall pathologic stage, age, histologic type, primary neoplasm focus type and neoplasm location. The K-M curve analysis confirmed that APOL1 expression was associated with OS in THCA. Furthermore, K-M analysis indicated that higher APOL1 levels were associated with improved prognosis in all subgroups except for patients with pathologic stage I, highlighting the potential of APOL1 as a diagnostic biomarker for distinguishing THCA from normal tissues. However, survival analysis revealed that the correlation between APOL1 upregulation and prognosis in certain THCA subtypes was not statistically significant, indicating the limited predictive power of APOL1 in these specific clinical contexts, consequently, additional clinical studies are required to further substantiate the prognostic significance of APOL1 in THCA.

To develop a more precise prognostic model, a total of seven genes co-expressed with APOL1 were identified using LASSO regression analysis and multifactorial regression analysis. AUC values and K-M analyses indicate that TRIM21 acts as a protective factor, while TRIM16L serves as a risk factor in THCA. Based on Cox regression analysis of clinical features and data from APOL1, TRIM16L and TRIM21, a nomogram was constructed to predict the 1-, 3- and 5-year OS. The calibration chart confirmed that APOL1 expression scores could predict OS, validating the prognostic significance of APOL1. Consequently, APOL1 may represent a novel prognostic biomarker in patients with THCA.

The biological functions of APOL1 in PTC were next assessed using GO and KEGG enrichment analysis. The analyses indicated that genes positively correlated with APOL1 expression are potentially involved in regulating immune functions within tumor tissues, whereas negatively correlated genes may influence tumor cell metabolism and proliferation. In the present study, it was shown that APOL1 functioned as a tumor suppressor in THCA, whereas previous studies have identified APOL1 as an oncogene that promotes proliferation and inhibits apoptosis through the NOTCH1 signaling pathway (21). The role of APOL1 in various tumors may depend on its tissue-specific expression and interactions with distinct molecular pathways. Tumor types differ in their microenvironmental and metabolic characteristics, which may influence APOL1 function. For example, in PTC, APOL1 may act as a tumor suppressor by regulating pathways that inhibit cell proliferation or promote apoptosis. Therefore, in different tissues, differences in interferon levels in the microenvironment, immune cell infiltration status, and dominant signaling pathways may determine the directionality of APOL1 function. Additionally, APOL1 triggers fibroblast proptosis in ulcerative colitis through the NLRP3/Caspase-1/GSDMD signaling pathway by releasing the chemokine CXCL1 (32). KEGG enrichment analysis showed that APOL1 was positively correlated with immune system processes, cytokine signaling, and Th1 and Th2 cell differentiation. Consequently, it was hypothesized that APOL1 primarily influenced THCA progression through the TME, positioning APOL1 as a potential biomarker and a prospective therapeutic target in THCA.

The immune surveillance model, which posits that immune cells can recognize and eliminate tumor cells, is well established (33). Tumors can evade immune destruction through mechanisms of elimination, homeostasis and escape, often leading to rapid tumor progression post-immune evasion (34, 35). Studies have demonstrated that ICI and the TME are crucial in tumor development, progression, prognosis and response to therapy (36–38). The role of APOL1 in the THCA TME has not yet been reported, to the best of our knowledge. Consequently, ESTIMATE and ssGSEA were used to uncover the correlation between APOL1 expression and ICI in THCA. The findings indicated that APOL1 expression correlates with ICI levels, including those of macrophages, neutrophils, DCs, T cells, Th1/2, CD8+ T cells, Tregs and B cells. The critical role of macrophage polarization has been elucidated in the development of oral squamous cell carcinoma as well as colorectal and gastric cancers (39–41). T cells are crucial for immune responsiveness, capable of activating and differentiating into subsets such as Th1, Th2, Th17 and Treg cells. Th1 promotes naive CD8+ T cell differentiation into cytotoxic T lymphocytes and enhances the cytotoxic activities of CD8+ T and NK cells (42–45). Perforin and granzyme, secreted by CD8+ T and NK cells, kill tumor cells through cytotoxicity. Additionally, NK cells can trigger tumor cell apoptosis via TNF-related apoptosis-inducing ligand (46). Th1, CD8+ T and NK cells are the principal effector cells against tumors. Herein, APOL1 expression was positively associated with infiltration of various immune cell types, including macrophages, neutrophils, DCs, T and B cells, Th1/2, CD8+ T and Tregs. This data suggests that APOL1 expression may be relevant for immunotherapeutic approaches in THCA, offering promising insights into the immune response dynamics of patients with THCA.

The effectiveness of immunotherapy relies upon adequate ICI into the TME alongside sufficient immune checkpoint expression (47). Increased APOL1 levels were associated with heightened expression of four immune checkpoint genes (HAVCR2, LAG3, PDCD1 and SLGLEC15), demonstrating a significant positive correlation between APOL1 and these checkpoints in THCA. Analysis indicated strong correlations between APOL1 and immune checkpoints, suggesting APOL1 as a potential immunotherapeutic target in THCA. Despite these promising results, the present study has limitations. APOL1 expression and its biological role were assessed using patient databases and cultured cells rather than *in vivo* models or *ex vivo* tissues. Additionally, each type of immune cell exhibits distinct, sometimes opposing functions in the TME. Further research is required to elucidate the specific role of APOL1 specific role and the underlying mechanisms in governing cell invasion and the TME in THCA.

In this study, overexpression of APOL1 is identified in THCA; however, its high expression paradoxically correlates with a favorable prognosis. This apparent contradiction can be elucidated through several mechanisms. First, the biological functions of APOL1 are significantly influenced by the cellular genetic background and the status of specific signaling pathways (45, 48). Intertumoral heterogeneity may also lead to divergent roles of APOL1 within the same cancer or cell type, further complicating its involvement in tumor progression (21, 49). Additionally, the role of APOL1 may be time-dependent; it could inhibit cell proliferation during certain stages of tumor development, while playing a more complex role in invasion and metastasis at later stages (49, 50). Lastly, the function of APOL1 may vary depending on the tumor microenvironment and cell type (51, 52), as APOL1 interacts with different molecules in different microenvironments, potentially exhibiting diverse functions. Consequently, the high expression of APOL1 and its association with a favorable prognosis in thyroid cancer does not contradict its oncogenic potential, but rather highlights its complex and multifaceted regulatory roles in tumor progression.

Our study is the first to comprehensively evaluate its function in THCA, and we innovatively explored the potential association between APOL1 and the immune microenvironment, and provided phenotypic evidence supporting its functional role in THCA. Our findings suggest that APOL1 may serve as a promising therapeutic target for the treatment of PTC. Nonetheless, this study has certain limitations. Notably, it did not include analyses of medullary thyroid carcinoma, nor did it investigate the effects of APOL1 on specific immune cell populations or tertiary lymphoid structures within the tumor microenvironment. The underlying molecular mechanisms through which APOL1 exerts its influence remain to be elucidated. These aspects will be prioritized in our future research to deepen our understanding of APOL1’s role in thyroid tumor immunobiology.

In summary, it was demonstrated that APOL1 expression is upregulated in THCA tissues. Knockdown of APOL1 in THCA cells results in promoted proliferation and migration. Additionally, APOL1 may regulate ICI in the THCA microenvironment.

## Data Availability

The original contributions presented in the study are included in the article/**Supplementary Material**. Further inquiries can be directed to the corresponding author.
