# Supplementary figures and images for: APOL1 is a novel prognostic biomarker in thyroid cancer and correlates with immune infiltration
Source: Front Oncol. 2025 Nov 25;15:1707078. doi: 10.3389/fonc.2025.1707078 (PMC12685650; doi:10.3389/fonc.2025.1707078)

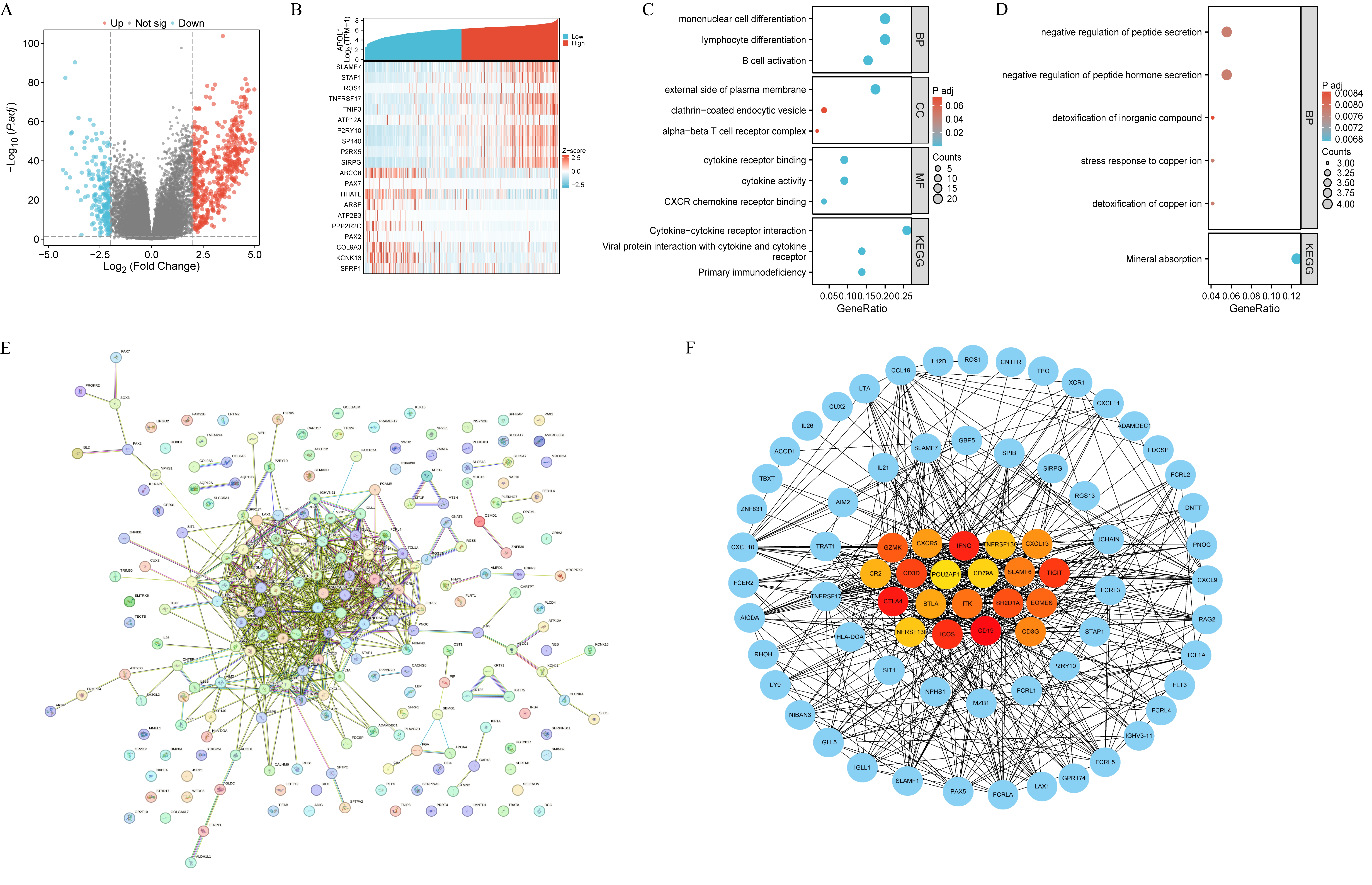

Supplement: Supplementary file 1 [file Image1.jpeg]
