# Supplementary material for: APOL1 is a novel prognostic biomarker in thyroid cancer and correlates with immune infiltration
Source: Front Oncol. 2025 Nov 25;15:1707078. doi: 10.3389/fonc.2025.1707078 (PMC12685650; doi:10.3389/fonc.2025.1707078)
Supplement: Supplementary file 2 [file Table1.docx]

Table S1 Clinical baseline information

| Characteristics | N | OR (95%CI) | P |
| --- | --- | --- | --- |
| Age(＞45 vs. ≤45) | 25 | 0.364(0.053-2.496) | 0.314 |
| Gender（Male vs. female） | 25 | 1（0.178-5.632） | 0.256 |
| Tumor location（Unilateral vs bilateral） | 25 | 3.333（0.599-18.543） | 0.175 |
| The number of lymph node metastases（＞4 vs.≤4） | 25 | 2.240（0.451-11.114） | 0.341 |
| Pathological stage（T1 vs.T2&T3&T4） | 25 | 8.571（0.825-89.040） | 0.119 |
| Extrathyroidal extension（Yes vs. NO） | 25 | 6.750（1.162-39.200） | 0.027 |
|  |  |  |  |
