# Supplementary material for: APOL1 is a novel prognostic biomarker in thyroid cancer and correlates with immune infiltration
Source: Front Oncol. 2025 Nov 25;15:1707078. doi: 10.3389/fonc.2025.1707078 (PMC12685650; doi:10.3389/fonc.2025.1707078)
Supplement: Supplementary file 3 [file Table2.docx]

Table S2. Univariate logistic regression

| Characteristics | n | Odds ratio (95% confidence interval) | P-value |
| --- | --- | --- | --- |
| Pathological T stage, T2 and T3 and T4 vs. T1 | 510 | 0.807 (0.548-1.189) | 0.279 |
| Pathologic N stage, N1 vs. N0 | 462 | 1.874 (1.295-2.713) | <0.001^c^ |
| Pathological M stage, M1 vs. M0 | 295 | 0.612 (0.161-2.328) | 0.472 |
| Pathological stage, Stage II and Stage III and Stage IV vs. Stage I | 510 | 0.638 (0.449-0.908) | 0.013^a^ |
| Sex, male vs. female | 512 | 0.906 (0.614-1.338) | 0.619 |
| Age, >45 vs. ≤45 | 512 | 0.614 (0.433-0.871) | 0.006^b^ |
| Histological type, Follicular and Tall Cell and Other vs. Classical | 512 | 0.388 (0.260-0.579) | <0.001^c^ |
| Residual tumor, R1 and R2 vs. R0 | 450 | 1.522 (0.867-2.670) | 0.143 |
| Extrathyroidal extension, Yes vs. No | 494 | 1.444 (0.984-2.118) | 0.060 |
| Primary neoplasm focus type, Unifocal vs. Multifocal | 502 | 0.658 (0.462-0.936) | 0.020^a^ |
| Neoplasm location, Left lobe and Right lobe vs. Bilateral and Isthmus | 506 | 0.590 (0.384-0.907) | 0.016^a^ |
| Thyroid gland disorder history, Lymphocytic Thyroiditis and Nodular Hyperplasia and Other, specify vs. Normal | 454 | 0.828 (0.565-1.212) | 0.331 |

^a^P<0.05, ^b^P<0.01, ^c^P<0.001.
