# Supplementary material for: APOL1 is a novel prognostic biomarker in thyroid cancer and correlates with immune infiltration
Source: Front Oncol. 2025 Nov 25;15:1707078. doi: 10.3389/fonc.2025.1707078 (PMC12685650; doi:10.3389/fonc.2025.1707078)
Supplement: Supplementary file 4 [file Table3.docx]

Table S3 Baseline data

| Characteristics | Low expression of APOL1 | High expression of APOL1 | P value |
| --- | --- | --- | --- |
| n | 256 | 256 |  |
| Pathologic T stage, n (%) |  |  | 0.255 |
| T1 | 66 (12.9%) | 77 (15.1%) |  |
| T2 | 94 (18.4%) | 75 (14.7%) |  |
| T3 | 82 (16.1%) | 93 (18.2%) |  |
| T4 | 13 (2.5%) | 10 (2%) |  |
| Pathologic N stage, n (%) |  |  | < 0.001 |
| N0 | 127 (27.5%) | 102 (22.1%) |  |
| N1 | 93 (20.1%) | 140 (30.3%) |  |
| Pathologic M stage, n (%) |  |  | 0.700 |
| M0 | 124 (42%) | 162 (54.9%) |  |
| M1 | 5 (1.7%) | 4 (1.4%) |  |
| Pathologic stage, n (%) |  |  | 0.003 |
| Stage I | 130 (25.5%) | 158 (31%) |  |
| Stage II | 38 (7.5%) | 14 (2.7%) |  |
| Stage III | 57 (11.2%) | 56 (11%) |  |
| Stage IV | 30 (5.9%) | 27 (5.3%) |  |
| Gender, n (%) |  |  | 0.619 |
| Female | 184 (35.9%) | 189 (36.9%) |  |
| Male | 72 (14.1%) | 67 (13.1%) |  |
| Race, n (%) |  |  | 0.093 |
| Asian | 18 (4.3%) | 33 (7.9%) |  |
| Black or African American | 16 (3.8%) | 11 (2.6%) |  |
| White | 165 (39.7%) | 173 (41.6%) |  |
| Age, n (%) |  |  | 0.006 |
| <= 45 | 106 (20.7%) | 137 (26.8%) |  |
| > 45 | 150 (29.3%) | 119 (23.2%) |  |
| Histological type, n (%) |  |  | < 0.001 |
| Classical | 159 (31.1%) | 207 (40.4%) |  |
| Follicular | 82 (16%) | 19 (3.7%) |  |
| Tall Cell | 10 (2%) | 26 (5.1%) |  |
| Other | 5 (1%) | 4 (0.8%) |  |
| Residual tumor, n (%) |  |  | 0.282 |
| R0 | 196 (43.6%) | 196 (43.6%) |  |
| R1 | 22 (4.9%) | 32 (7.1%) |  |
| R2 | 1 (0.2%) | 3 (0.7%) |  |
| Extrathyroidal extension, n (%) |  |  | 0.060 |
| No | 179 (36.2%) | 161 (32.6%) |  |
| Yes | 67 (13.6%) | 87 (17.6%) |  |
| Primary neoplasm focus type, n (%) |  |  | 0.020 |
| Multifocal | 103 (20.5%) | 130 (25.9%) |  |
| Unifocal | 147 (29.3%) | 122 (24.3%) |  |
| Neoplasm location, n (%) |  |  | 0.054 |
| Bilateral | 38 (7.5%) | 50 (9.9%) |  |
| Isthmus | 6 (1.2%) | 16 (3.2%) |  |
| Left lobe | 95 (18.8%) | 83 (16.4%) |  |
| Right lobe | 115 (22.7%) | 103 (20.4%) |  |
| Thyroid gland disorder history, n (%) |  |  | < 0.001 |
| Lymphocytic Thyroiditis | 28 (6.2%) | 46 (10.1%) |  |
| Nodular Hyperplasia | 50 (11%) | 18 (4%) |  |
| Normal | 138 (30.4%) | 148 (32.6%) |  |
| Other, specify | 11 (2.4%) | 15 (3.3%) |  |
