# Supplementary material for: APOL1 is a novel prognostic biomarker in thyroid cancer and correlates with immune infiltration
Source: Front Oncol. 2025 Nov 25;15:1707078. doi: 10.3389/fonc.2025.1707078 (PMC12685650; doi:10.3389/fonc.2025.1707078)
Supplement: Supplementary file 5 [file Table4.docx]

Table S4. COX regression of seven genes screened by LASSO.

| Characteristic | n | Univariate analysis | | Multivariate analysis | |
| --- | --- | --- | --- | --- | --- |
|  |  | Hazard ratio (95% CI) | P value | Hazard ratio (95% CI) | P value |
| APOL1 | 512 |  | 0.016^a^ |  | 0.335 |
| Low | 256 | Reference |  | Reference |  |
| High | 256 | 0.211 (0.060-0.744) |  | 0.512 (0.131-1.995) |  |
| TRIM21 | 512 |  | 0.006^b^ |  | 0.022^a^ |
| Low | 256 | Reference |  | Reference |  |
| High | 256 | 0.126 (0.028-0.554) |  | 0.163 (0.035-0.768) |  |
| TRIM16L | 512 |  | 0.039^a^ |  | 0.089 |
| Low | 256 | Reference |  | Reference |  |
| High | 256 | 3.303 (1.062-10.275) |  | 2.749 (0.856-8.827) |  |
| UNC13D | 512 |  | 0.949 | - |  |
| Low | 256 | Reference |  | - |  |
| High | 256 | 0.969 (0.363-2.583) |  | - |  |
| HDHD2 | 512 |  | 0.215 | - |  |
| Low | 256 | Reference |  | - |  |
| High | 256 | 1.898 (0.689-5.227) |  | - |  |
| PEG3 | 512 |  | 0.524 | - |  |
| Low | 256 | Reference |  | - |  |
| High | 256 | 1.379 (0.513-3.708) |  | - |  |
| NOD2 | 512 |  | 0.304 | - |  |
| Low | 256 | Reference |  | - |  |
| High | 256 | 1.702 (0.618-4.692) |  | - |  |

^a^P<0.05, ^b^P<0.01. CI, confidence interval; LASSO, Least Absolute Shrinkage and Selection Operator; APOL1, apolipoprotein 1; TRIM21, Tripartite Motif Containing 21; TRIM16L, Tripartite Motif Containing 16 Like; UNC13D, Unc-13 Homolog D; HDHD2, Haloacid Dehalogenase Like Hydrolase Domain Containing 2; PEG3, Paternally Expressed 3; NOD2, Nucleotide Binding Oligomerization Domain Containing 2.
